# Do Anthropometric Health Risk Indicators of South African Primary School Children Require National Growth Charts? Insights from the NW-CHILD Study

**DOI:** 10.3390/children13030372

**Published:** 2026-03-06

**Authors:** Xonné Muller, Anita E. Pienaar, Barry Gerber, Naomi E. Brooks, Danita Kruger, Colin N. Moran

**Affiliations:** 1 Physical Activity, Sport and Recreation Focus Area, Faculty of Health Science, North-West University, Potchefstroom 2520, South Africa; anita.pienaar@nwu.ac.za (A.E.P.); barry.gerber@nwu.ac.za (B.G.); 2 Department of Human Movement Sciences, University of Fort Hare, Alice 5700, South Africa; 3 Faculty of Health Sciences and Sport, University of Stirling, Scotland FK9 4LA, UK; n.e.brooks@stir.ac.uk (N.E.B.); colin.moran@stir.ac.uk (C.N.M.); 4 Department of Kinesiology, DePauw University, Greencastle, IN 46135-0037, USA; 5 Focus Area for Pure and Applied Analytics, Faculty of Natural and Agricultural Sciences, North-West University, Potchefstroom 2531, South Africa; danita.kruger@nwu.ac.za

**Keywords:** body composition, profiles, primary school children, growth charts, growth monitoring

## Abstract

**Highlights:**

**What are the main findings?**

- Age, gender, and socioeconomic status (SES) significantly influence height, body mass index (BMI), waist circumference, and skeletal muscle in South African primary school children.
- South African children from low SES aligned closely with World Health Organization (WHO) standards at the 50th percentile, whereas children from high SES trend above these expected standards.

**What are the implications of the main findings?**

- Anthropometric cut-points are needed that extend beyond height, weight, and BMI for primary school children in South Africa (SA), incorporating the influence of age, gender, and SES.
- Nationally developed growth references taking into account age, gender, and SES are needed to timeously and comprehensively screen for health risks in South African primary school children.

**Abstract:**

Background/Objectives: Growth monitoring and screening are vital indicators for child wellness. Controversy exists regarding the use of national versus international growth charts for school-going children. This study investigated the suitability of existing global references, considering the influence of age, gender, and socioeconomic status (SES), and the specific growth patterns across multiple anthropometric health indicators (AHIs). Methods: A total of 349 children (boys = 165, girls = 184, low SES = 201, high SES = 148) were measured longitudinally at ages 6, 9, and 12 years while attending primary school. AHI included stature, body mass, body mass index (BMI), body fat percentage (BFP), skinfold thickness, skeletal muscle, and waist circumference (WC). Results: Three-way interaction effects were found for age, gender, and SES on WC and skeletal muscle (*p* = 0.05). Several two-way interactions emerged for age and gender (height, BMI, skeletal muscle; *p* < 0.01), as well as age and SES (height, BMI, skeletal muscle; *p* < 0.01) and gender and SES (height, skeletal muscle, WC, *p* < 0.05). Cut-points for height, body mass, and BMI at the median, compared to universal standards, indicate that the total group fell at, or slightly above, World Health Organization (WHO) standards. The low SES group aligned with WHO standards, whereas the high SES group trended above the reference values at most points. Conclusions: The WHO growth standards are suitable for general monitoring in South African children aged 6–12 years but provide limited context-specific interpretation across age, gender, and socioeconomic backgrounds. National age-, gender-, and SES-specific growth references, incorporating additional anthropometric indicators, are needed to support locally relevant screening.

## 1. Introduction

For decades, growth monitoring and screening have been recognised as the gold standard for assessing healthy growth and physical development in children [1,2,3]. Several anthropometric health indicators (AHI), including height-for-age, weight-for-age, weight-for-height, and BMI-for-age, are used as toolkits to assess child nutritional status and growth [3,4,5,6,7]. Undernutrition (wasting, stunting, and underweight), hidden hunger (an imbalance in essential nutrients), and overnutrition (overweight and obesity) are vital nutritional indicators for development [4,5] and are defined as falling below or above certain AHI cut-offs.

Children may experience one or more of these conditions simultaneously [5,6,7]. This is known as the double or triple burden of malnutrition (DBM). Such malnutrition is a global health epidemic [5] rooted in poverty as the leading cause [8,9] and has inverse effects on the growth and development of children [4,5]. This is most evident in lower-middle-income countries (LMICs) such as Russia, Brazil, and South Africa [6,7]. Several health and developmental risks, such as NCDs, poor academic achievement, and impaired motor performance, are among the adverse outcomes linked to impaired growth and development [4,5,6,8,10,11,12,13].

Over the past decade, obesity, previously linked to high socioeconomic status (SES) groups, has become more prevalent in low SES groups and rural communities [9]. Consequently, after an extensive review by the National Center for Health Statistics/World Health Organization (NCHS/WHO), revised growth references were recommended by the WHO in 2006 [1,14]. Since linear growth is known to be affected by various factors, including poor nutrition, hereditary predispositions, and environmental factors [1,15], these newly developed growth charts included children from various SES backgrounds and continents, including South America, Africa, Asia, Europe, the Middle East, and North America, making them more representative These reference datasets are considered the gold standard for screening and monitoring populations [16,17]. However, they are not universally accepted [18,19,20,21].

There is an ongoing debate over the use of national versus international standards, given that genetic and population-specific differences [16,19,20,21,22,23,24,25], ethnicity, and SES can influence outcomes [1,18]. In addition, epigenetic and intergenerational effects on child growth make optimal growth unlikely even for those raised in advantaged conditions or high-income countries [20]. Several countries, such as China, Korea, and Finland, have already developed their own national growth references due to concerns that the WHO references do not adequately account for population and environmental diversity [16,19,22,24,25]. Differences in the timing of growth and peak height velocity (PHV) across genders and populations, as well as within SES groups, have also been identified [26,27,28,29]. Researchers found that weight trajectories of boys and girls also deviate from WHO curves compared to national references [25,30].

Because of the strict inclusion criteria of the revised WHO references, they are unlikely to represent the behavioural, social, genomic, and environmental diversity of their respective nations [20]. These discrepancies between national and international growth references suggest the need for further investigation, as SES, ethnicity, gender, environment, and genetics may influence growth and development [23,27]. National bodies should advise on the use of national or international growth references for assessing and regularly tracking growth in children and young adults, given population diversity [27,31]. This will facilitate the identification of health risk indicators and secular trends in growth and development within a nation [27].

South Africa (SA) lacks national growth charts for reference, which could be problematic, given that SA is an SES-diverse country and includes several ethnic populations [22,32,33,34,35,36]. Malnutrition in the form of stunting and overweight/obesity (OW/OB) is a serious health concern among the South African child population. Although several researchers have investigated growth in the South African context, much of the work is outdated [35,36] or focuses mainly on malnutrition indicators [36,37,38], or the relationship between growth and factors like physical fitness, motor skills, gender, cognitive development, and poverty [39,40,41,42,43]. Most studies to date have examined children under 6 or adolescents aged 13–15 years, often using cross-sectional or short-term data, and have not included both urban and rural populations [41,43].

This study aimed to determine whether distinct growth patterns emerge across AHI levels and whether SES and gender are associated with these patterns in apparently healthy South African children over the long term. The study further investigated the applicability of the WHO reference growth curves for stature, body mass, and BMI to determine the necessity of national growth charts. This investigation spans over three time-points of seven school years, focusing on primary school children aged 6 to 13 years from the North-West Province (NWP) of SA. The results of this investigation will serve as a valuable preliminary pilot, highlighting observable differences to inform and motivate policy development in SA. The findings can potentially also serve as a valuable toolkit for monitoring growth trajectories and guiding screening for health risks in children throughout primary schools in SA, as part of assessing the effectiveness of intervention strategies to ensure normal growth and development.

## 2. Materials and Methods

### 2.1. Research Design

**Figure 1:**
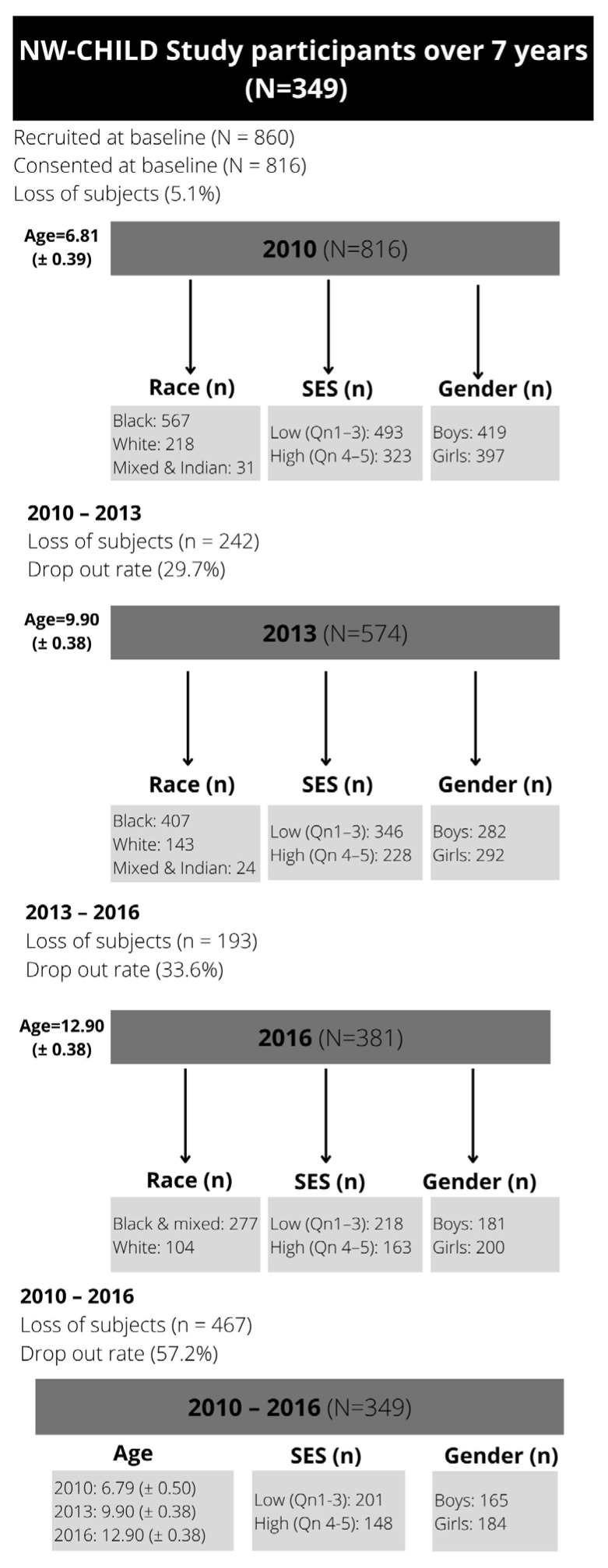
NW-CHILD study participants (2010–2016).

This longitudinal study is a sub-study of the NW-CHILD (Child Health Integrated Learning and Development) study that was conducted from 2010 to 2016 in the NWP of SA. The study included three time point measurements, with baseline measurements in 2010 and two follow-up measurements in 2013 and 2016, which spanned the seven primary school years (Figure 1). The NWP, home to approximately 8% of SA’s population, is characterised by high poverty levels (59.6%), highlighting its dire circumstances compared to the national poverty rate of 40% [44,45]. Children aged 6 to 13 were recruited via random, stratified sampling, with selection based on gender, school quintile, and four school districts in the NWP. Additionally, five schools were identified in each of the four school districts, totalling 20 schools to be included in the study for testing purposes. These schools were also classified by school quintile (1–5) according to SES, with students from low to middle SES (quintiles 1–3) and high SES (quintiles 4 and 5) in each district [46]. The identified children were tested in Grade 1 (2020), Grade 4 (2013), and Grade 7 (2016).

A detailed illustration of the participants, categorised by gender and SES classification, was provided in a previous study by Muller et al. [47], including mean ages and the number of participants at each time point. Participant dropout was treated as missing at random and did not have a significant impact on the data. No bias was reported, as noted in a previous study of the NW-CHILD sample by Pienaar [48].

### 2.2. Ethical Considerations

The NW-CHILD study received ethical clearance from the Health Research Ethics Committee (HREC) of North-West University (NWU) (00070-09-A1) and approval from the North-West DBE. Ethical clearance specific to this sub-study was also obtained (00070-09-A1-04). All school principals granted consent for conducting tests during school hours, and parents were provided with informed consent forms. Children under 8 years of age gave assent, while older children provided consent to participate in the study, provided their parents had also given their consent. Re-consent was also provided during follow-up testing in 2013 and 2016.

### 2.3. Measurement Instruments and Apparatus

#### 2.3.1. Anthropometric Measurements

All children were measured for the following anthropometric assessments by qualified Level 2 Kinantropometrists according to the International Society for the Advancement of Kinanthropometry (ISAK) guidelines [49]: stature (cm), body mass (kg), skinfolds (subscapular, calf, and triceps) (mm), body fat percentage (BFP) (%), relaxed upper arm and waist circumferences (WC) (cm). Height and body mass were measured using a Harpenden portable stadiometer (Holtain Limited, Crymych, UK) and an electronic scale (BF 511, Omron, Kyoto, Japan), to the nearest 0.1 cm and 0.1 kg. For BMI calculations, stature and mass measurements were used (kg/m^2^). Body compositions (fat mass and skeletal muscle) were measured with the BF 511 Omron bio-impedance analyser (Omron, Kyoto, Japan). Skinfolds and circumferences were measured twice using a metal measuring tape (Cescorf, Porto Alegre, Brazil) and a Harpenden skinfold calliper to obtain the average to the nearest 0.1 mm, ensuring validity and reliability while accounting for the technical error of measurement. All measurements were taken in private and with minimal clothing.

#### 2.3.2. Anthropometric Health Indicators (AHI)

BMI was used to define cut points and assign AHI categories, specifically for thin, overweight, and normal-weight individuals, taking into account gender and age [50,51]. A low weight-for-age indicates thinness [51]. Because weight-for-age z-scores are not recommended and not available in the WHO Anthroplus software programme (2022) for children older than 10, thinness is used as an indicator of underweight, which is similar to the WHO standards. Subsequently, as weight-for-age z-scores do not differentiate between height and body weight, their interpretation requires caution [45]. Further reasoning is discussed in a previous study by Haywood and Pienaar [46]. BMI percentiles specific to age and gender were used to assess the severity of thinness, categorised into grades 1 to 3: grade 1 < 18.5 (moderate), grade 2 < 17 (average), and grade 3 < 16 (severe) [50,51]. Stunting was determined by height-for-age z-scores (HAZ) according to the WHO cut-points of −2 z-score or greater, calculated by Antrhoplus (2022) [3,46,52]. The cut-points established by Lohman [53] were used to calculate the combined measurement of two skinfolds (triceps and subscapular), which are reported to be most closely associated with body fat in children [54]. These age- and gender-specific cut-points classify children as normal weight (on or above the 50th percentile), overweight (on or above the 85th percentile), and obese (on or above the 95th percentile) [53]. Similarly, body fat percentage (BFP) as a measurement of skinfolds is classified according to age- and gender-specific categories at the 50th, 85th and 95th percentiles according to cut-points by Lohman and Going [55].

#### 2.3.3. Statistical Analysis

Descriptive statistics (means, standard deviations, minimum and maximum ranges) over three time points (2010–2016) for all anthropometric variables were analysed through the R software Package, version 4.4.2 [56]. Mixed linear growth curve models were utilised to analyse the trajectories of growth and AHI over the 7-year timespan. The AHI was used as the response variable, with the time points of 2010, 2013, and 2016 regarded as fixed effects. To assess the assumptions of normality, homoscedasticity, linearity, independence of errors, and the absence of outliers, plots of standardised residuals were employed. Percentiles, indicating the ranges for AHI, were determined using the LMS (Lambda, Mu, Sigma) method of Cole and Green (1992) [57]. LMS defines the conversion of skewness (L), median (M), and coefficient of variation (S). The applicability of WHO growth references was assessed descriptively by examining percentile distributions and z-score patterns across age, gender and SES, without formally testing the magnitude of deviations for statistical significance due to lack of access to raw data.

## 3. Results

A total of 349 participants, 165 boys and 184 girls, were included in this study from both high (boys = 82; girls = 66) and low (boys = 83; girls = 118) SES. Quintile 1–3 schools represented low SES and included 58% (*n* = 201) of the sample, and quintile 4–5 schools 42% (*n* = 148). The total sample had a mean age of 6.90 ± 0.50 years at baseline in 2010 (Grade 1, T1), 9.90 ± 0.38 years in 2013 (Grade 4, T2), and 12.90 ± 0.38 years in 2016 (Grade 7, T3). The study will refer to the mean ages of 6, 9, and 12 during the reporting and discussion of the results.

**Figure 2:**
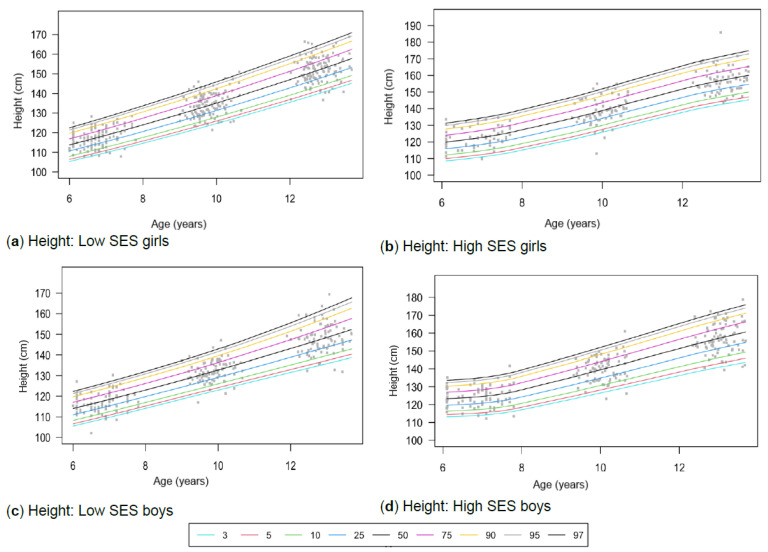
(**a**–**d**) Growth curve comparison for height according to gender and SES.

**Figure 3:**
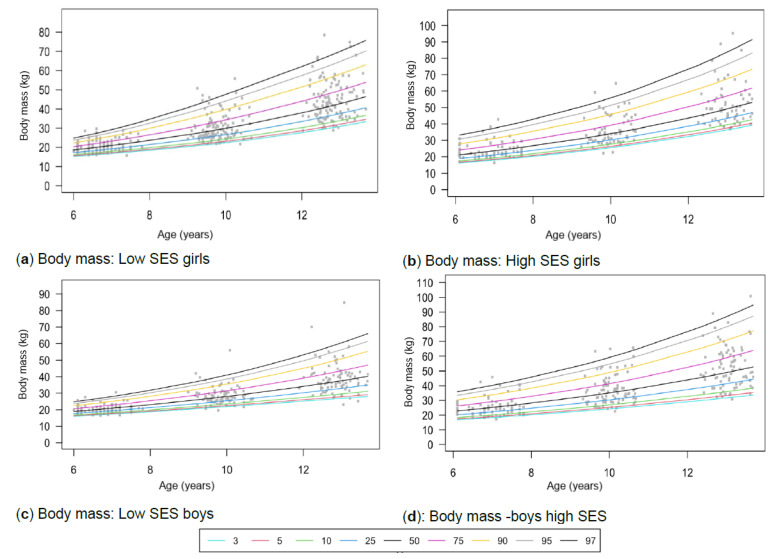
(**a**–**d**) Growth curve comparison for body mass according to gender and SES.

**Figure 4:**
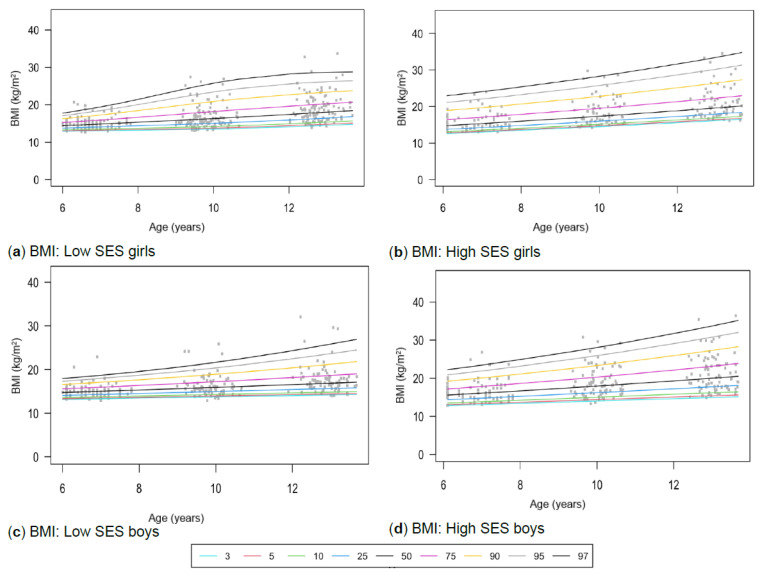
(**a**–**d**) Growth curve comparison for BMI according to gender and SES.

**Figure 5:**
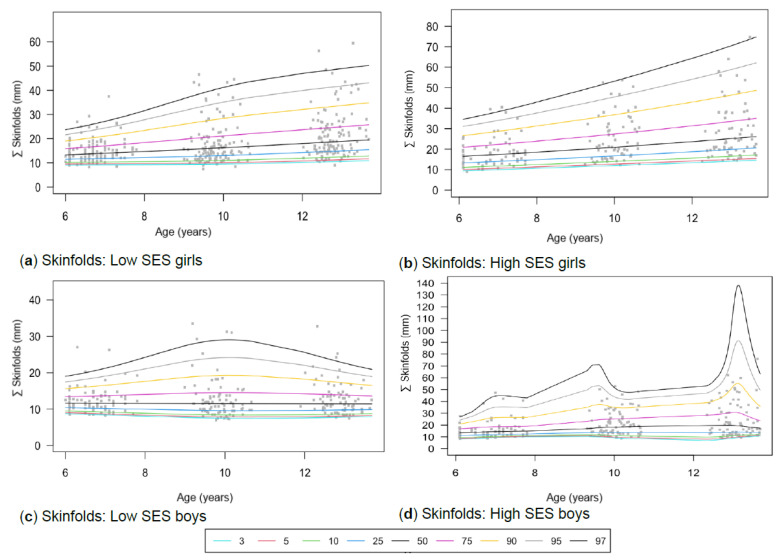
(**a**–**d**) Growth curve comparison for ∑skinfolds according to gender and SES.

**Figure 6:**
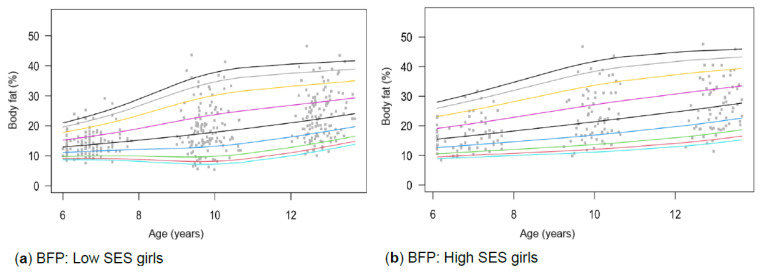

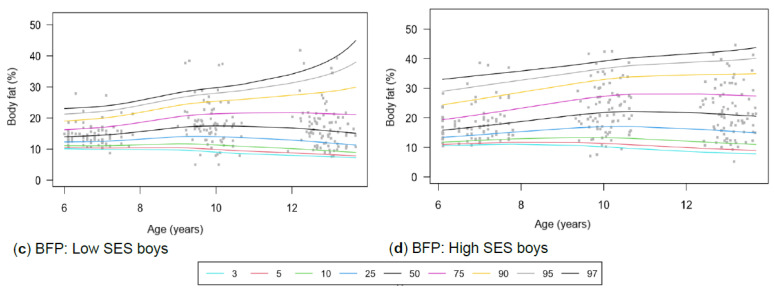
(**a**–**d**) Growth curve comparison for body fat according to gender and SES.

**Figure 7:**
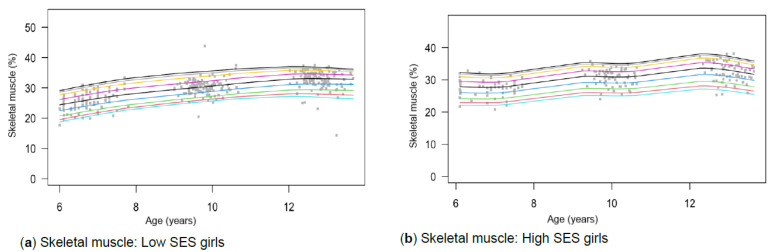

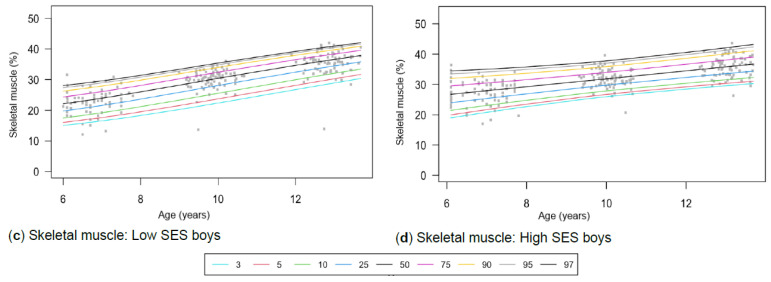
(**a**–**d**) Growth curve comparison for skeletal muscle according to gender and SES.

**Figure 8:**
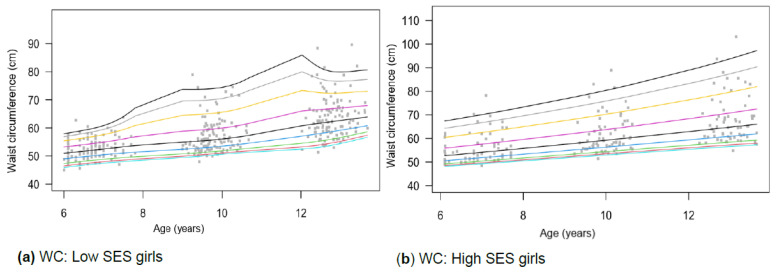

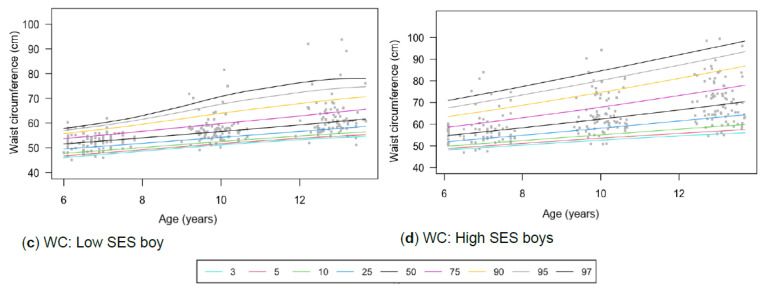
(**a**–**d**) Growth curve comparison for waist circumference according to gender and SES.

Table 1 provides the descriptive characteristics for age and body composition (body mass, height, body fat percentage, sum of skinfolds, BMI, percentage skeletal muscle and WC) and z-scores (HAZ, WAZ, BAZ) of the total group (*N* = 349) for each time point measurement as per gender and divided into SES (high, low) groups. Children from high SES were consistently taller by at least 5 cm compared to low SES groups, with greater discrepancies noticeable among boys (8 cm) (*p* < 0.05). Similar trends were observed for body mass, with greater disparities between the SES groups as children aged, ranging between approximately 4–8 kg in girls and 6–13 kg in boys over the 7-year timespan. Differences in stature and body mass were further noticeable, as boys from low SES were slightly shorter and weighed less (skinnier/thinner) at all time points compared to boys from high SES and girls (low and high SES). SES differences where body composition measurements were greater in children from high SES were also noted in most of the other anthropometric measurements, except for skeletal muscle mass at age 12, where boys (high: 35.91% ± 3.40; low: 35.90% ± 3.40) and girls (high: 32.7% ± 3.10; low: 32.50% ± 3.00) from both SES groups had slightly similar skeletal mass. Notable gender differences also emerged at this age, indicating that boys in both low and high SES groups had a greater skeletal mass compared to girls. Table 2 and Figure 2a–d, Figure 3a–d, Figure 4a–d, Figure 5a–d, Figure 6a–d, Figure 7a–d and Figure 8a–d highlight these significant differences according to age, gender, and SES growth curve trends for AHI.

Regarding z-score indices, both SES and gender groups fell within normal ranges for HAZ and WAZ (i.e., less than 2 SD). Notably, boys from low SES experienced the greatest adverse impact from HAZ at age 12, with a mean of −0.97SD (±1.00) compared to all other groups. Similarly, at the age of 6, boys from low SES also had the lowest mean value of −0.67 SD (±0.87) for WAZ compared to the other SES and gender groups. All groups were within the normal range for BAZ (BAZ < 1 SD), with boys from low SES having the lowest BAZ scores (−0.47 SD ± 1.36), while boys from high SES had the highest BAZ scores (0.70 SD ± 1.44).

Table 2 shows significant three-way and two-way interaction effects of AHI with age, gender, and SES. Only two AHI, including skeletal muscle (*p* = 0.010) and WC (*p* = 0.047), showed significant three-way interaction effects between age, gender, and SES. Four AHI showed two-way interaction effects for age and SES (height, BMI, WC, and skeletal muscle), while three AHI showed two-way interactions for gender and SES (height, skeletal muscle, and WC). Additionally, three interaction effects were found for age and gender models (height, BMI, and skeletal muscle). Table 3 presents the percentile values for girls and boys in the respective graphs.

Additionally, Table 4 presents comparisons between the age- and gender-specific percentiles by SES at ages 6, 9, and 12 years in this study and the WHO cut-points, which are available only for body mass, height, and BMI. For comparison, the 50th percentile will be used primarily.

### 3.1. Height

Significant two-way interactions are observed for three models within the group comparison: age and gender (*p* < 0.01), age and SES (*p* < 0.01), and gender and SES (*p* = 0.02) (Table 2). Girls from high SES who fall within the 50th percentile are, on average, at least 3 cm taller compared to those from low SES (Table 3). Similarly, boys from high SES falling within the 50th percentile are at least 7 cm taller at all ages compared to those from low SES. Slight differences are noted in girls from high SES. In this regard, height trajectories seem to taper off (Figure 2b) compared to girls from low SES (Figure 2a) and boys (Figure 2c,d), where steep inclines in height are observed. As expected, stature increases with age.

Table 4 reveals that most of the groups (NW-T and NW-L) align with WHO references at the 50th percentile, except for the NW-H group, which exceeds these references by 5 cm at age 6 (115.13 cm; 120.50 cm) and 12 years (151.23 cm; 156.80 cm), and 3 cm at age 9 years (132.49 cm; 135.95 cm). Boys from high SES in the 50th percentile display similar trends that surpass the WHO references, with approximately 8 cm at age 6 years (115.95 cm; 123.70 cm), 7 cm at age 9 years (132.57 cm; 139.80 cm), and 5 cm at age 12 years (149.08 cm; 154.70 cm). Contrastingly, the low SES group for boys reached a stature similar to global references at the 50th percentile at age 6 years (115.10 cm), while from age 9 years (130.85 cm) to 12 years (147.50 cm), they fell slightly below global standards. Most groups also exceed the WHO reference values at the 3rd and 97th percentiles (Table 4).

### 3.2. Body Mass

Similar trends can be observed for both genders and SES concerning body mass in Figure 3a–d. This is confirmed by the insignificant interaction effects for body mass (Table 2). However, larger differences are observed among those at the 97th percentile, as shown in Table 3. No WHO reference data on body mass are available for 12-year-olds, as the WHO recommends using BMI, because height increases during puberty, which can be mistaken for excess body mass (Table 4). Therefore, comparisons could only be made at 6 and 9 years. At the 3rd and 97th percentiles, all groups have body mass values that are similar to or higher than the WHO reference. Concerning the 50th percentile, girls from the NW-T (6 years = 20.40 kg; 9 years = 29.00 kg) show compatible references that are aligned with the WHO standards (6 years = 20.16 kg; 9 years = 28.20 kg), while the boys show slightly higher body mass values (6 years = 21.30 kg; 9 years = 29.35 kg) than the WHO references. Additionally, girls in the NW-H group at the 50th percentile weigh at least 3 kg more at age 9 (31.65 kg), similar to NW-H boys, who weigh approximately 7 kg more at age 9 (35.80 kg). However, the NW-L girls (19.65 kg; 27.60 kg) and boys (19.80 kg; 27.30 kg) fell slightly below the percentile references at ages 6 and 9.

### 3.3. Body Mass Index (BMI)

Significant two-way interaction effects for BMI were found between age and gender (*p* < 0.01) and between age and SES (*p* < 0.01) (Table 2). Growth patterns differ notably across SES groups, with the low SES (Figure 4a,c) and high SES (Figure 4b,d) groups following distinct trajectories, particularly for those above the 50th percentile (Table 3). Girls and boys from high SES at the 97th percentile have higher average BMIs of 7 kg/m^2^ and 5 kg/m^2^, respectively, by age 12, compared with girls and boys from low SES at the same centile. Additionally, significant age and gender interaction effects are also noted, with girls surpassing the BMI values of boys at the age of 12.

All the groups (NW-T, NW-L and NW-H for boys and girls) have mostly similar BMI values compared to WHO references for the 3rd and 97th percentiles (Table 4). Exceptions are noticed for NW-H groups, where greater BMI values were evident at most time points. Similar trends are noticed for the 50th percentile, with the boys from high SES (16.00 kg/m^2^; 18.15 kg/m^2^; 20.80 kg/m^2^) at ages 6, 9 and 12 and girls at ages 9 and 12 (16.93 kg/m^2^; 19.80 kg/m^2^) exceeding the WHO cut-points (16.10 kg/m^2^; 18.00 kg/m^2^). In most cases where any of the groups (NW-T, NW-L and NW-H for boys and girls) surpassed the 97th BMI percentile, these groups were classified above the 99th percentile according to WHO cut-off points.

### 3.4. Skinfolds and Body Fat Percentage (BFP)

The sum of skinfolds shows distinct trajectories for gender and SES groups (Figure 5a–d), while distinct trajectories for boys (Figure 6a–d) are observed in BFP. However, no significant interaction effects were found for skinfolds or BFP.

### 3.5. Skeletal Muscle

A significant three-way interaction among skeletal muscle, age, gender, and SES was found (*p* < 0.01). Additionally, significant two-way interaction effects were found for all three models: age and gender (*p* < 0.01), age and SES (*p* < 0.01), and gender and SES (*p* = 0.01). Regarding the interaction between gender and SES, distinct trajectories in muscle growth can be observed for both groups (Figure 7a–d). Girls from low SES show a steady incline from the age of 6, tapering off at age 12 years (Figure 7a). In contrast, growth curves for girls from high SES show various inflexion points indicating increases, plateaus, and a decline from the age of 12 (Figure 7b). Nonetheless, girls from low and high SES backgrounds reach relatively similar values at the age of 12 years (Table 3). For boys, fairly similar trends are noted among the different SES groups (Figure 7c,d). Boys from low SES groups at the 50th percentile have lower skeletal muscle mass than those from high SES groups. However, with time, the gap lessens.

### 3.6. Waist Circumference (WC)

Three-way (*p* = 0.047) and two-way interaction effects for WC are reported for age and SES (*p* < 0.010) and gender and SES (*p* = 0.039) (Table 2). The high SES groups for boys (Figure 8d) and girls (Figure 8b) showed similar trajectories. However, distinct differences are observed between the lower SES groups. Growth trajectories for girls from low SES show several inflexion points. A more pronounced inflexion is observed in both genders among those above the 50th percentile (Figure 8a), with a 10 cm difference between SES groups at age 12, with individuals from high SES having greater WC (Table 3). In most cases, boys also have higher WC values than girls.

## 4. Discussion

This study aims to determine whether distinct growth patterns exist in body composition characteristics among children in the NWP of SA by comparing body composition between children from high and lowSES backgrounds, controlling for age and gender, using longitudinal data. Additionally, direct comparisons were made with WHO reference data available for height, body mass, and BMI. Considering the overall group of typically developing children on the median for height, body mass, and BMI, children from the NWP slightly surpassed the WHO standards, falling between the 50th and 75th percentiles at most ages. Further comparisons to WHO references at the 50th percentile indicate that those from high SES exceeded the references by a considerable margin, mostly falling at or above the 75th percentile. The low SES groups were mainly aligned with or slightly below WHO standards (within the 25th–50th percentiles) for height, body mass, and BMI at most ages. Boys from high SES showed greater discrepancies relative to WHO standards, placing them between the 85th and 95th percentiles at age 6, between the 85th–90th percentiles at age 9, and between the 75th–85th percentiles at age 12 for height. Regarding body mass, boys fell within the 90th–95th percentile at ages 6 and 9.

### 4.1. Socioeconomic Status (SES), Age, and Gender Differences in Anthropometric Health Indicators (AHI) Growth Patterns

The results revealed several significant interactions among AHI, age, gender, and SES, underscoring the need to consider sociodemographic factors in growth trajectories.

#### 4.1.1. Height

Increases in stature in line with ageing have been reported as expected (Table 1). Height showed significant interactions within the three models (Table 2), where boys and girls from high SES showed higher stature values (*p* < 0.05) (Table 1). Findings suggest that gender and SES influence height trajectories, shaping patterns of height development over time. Similarly to this study, Silva et al. [32] and Freitas et al. [58] reported that Brazilian and Portuguese children from higher SES groups have taller stature than those from lower SES groups. The 5 cm differences in girls and up to 8 cm in boys are consistent with results from Brazilian children, a LMIC, showing significant height differences between SES groups of 7- to 17-year-old boys (2.7–5.6 cm) and girls (3.1–5.3 cm) [32].

Slight SES differences are further noted in trajectories where the height increases in girls from high SES seem to taper off at the age of 12 years (Figure 2a), compared to girls from low SES who are still experiencing height increases at this age (Figure 2b). Girls, including those from low SES, were taller compared to boys from low SES at the age of 12. This could be attributed to girls experiencing an earlier growth spurt [59]. Similarly, girls from high SES may also reach puberty earlier compared to those from low SES, and in turn, low SES girls will still be in their adolescent growth spurt when high SES girls are already experiencing a plateau at age 12 [60]. Aurino et al. [59] report that SES can influence the timing of puberty, supporting the latter statement that children from low SES backgrounds reach adult height at later ages. This ultimately gives them an opportunity for catch-up growth (CUG) during puberty [61]. However, catch-up is only partial, and height differences remain noticeable among males and females from different SES settings after reaching adult stature at age 19 [59]. Growth and maturation may occur at different times across SES groups, making it risky to create separate growth profiles too early, which could be misleading. These factors complicate the development of unique growth curves across SES groups, and only longitudinal research can determine whether these differences persist over time.

#### 4.1.2. Body Mass

As expected, age-related differences were reported with increasing age (Table 1). However, no significant interaction effects emerged, which translated to similar growth curves for both gender and SES groups concerning body mass trajectories (Figure 3a–d). Although trajectories follow the same trend, SES differences are observed across gender groups. Other studies have also noted differences in body mass between SES groups, with children from high SES groups being heavier [58]. This could be attributed to children from low SES being exposed to food poverty and relying on feeding schemes at school as possibly the only meals for the day. In contrast, those from highSES backgrounds are exposed to poor diet quality, including limited access to nutrient-dense foods [4,62].

#### 4.1.3. Body Mass Index (BMI)

Since differences in stature and body mass were observed, it was expected that differences in BMI would also be evident in growth patterns for BMI between the SES and gender groups, especially when exceeding the 50th percentile (Figure 4a–d). Notably, mean BMI values of the low SES group at the third percentile also shifted from the age of 9 onward to levels below the WHO references (Table 4), confirming that the growth of these already vulnerable children is increasingly at risk with advancing age. It is therefore important to monitor this growth trend at older ages.

The current study also reported interaction effects between BMI and age, gender, and SES. Older children, children from high SES, and boys had higher BMI values than younger children (6- and 9-year-olds), girls, and children from low SES. Similar results were reported in a Chinese cohort of 7- to 17-year-old children, in which boys had slightly higher BMI values than girls [63]. In contrast to the Chinese cohort, American girls have higher BMI values from age 12 onward [64]. McCarthy et al. [65] also reported slightly higher BMI values from the age of 8 onwards in UK girls, agreeing with our findings that indicate slightly higher BMI values in girls at the age of 12. However, researchers noted that BMI does not accurately reflect body fat accumulation in children and therefore advocate the use of alternative body composition measures [64]. A German study also reports that low SES is mainly associated with higher BMI, which contrasts with our findings. However, these trends are more synonymous with first-world countries [66]. Nonetheless, the WHO prefers using BMI over body mass beyond the age of 10, as body mass can be influenced by factors such as skeletal muscle mass, especially during puberty [14]. Additionally, using BMI as a predictor of body fat has limitations, as BMI is not a strong indicator of total body fat and fails to account for relative body fat, particularly in children under 9 years old. It is also cautioned that BMI comparisons across ethnically diverse populations should be approached with caution due to differences in genetic makeup, body size, and proportional differences [23,67,68,69]. This questions the use of the same cut-point measurements for an ethnically diverse country such as South Africa.

#### 4.1.4. Body Fat Percentage (BFP) and Skinfold Thickness

Although no specific gender, age, or SES differences emerged from this study for skinfolds and body fat percentage, other researchers have developed national cut-points for skinfold thickness to assist with the early detection of obesity in children. This has been highlighted as an area of concern since there is no gold standard or national representative cut-points, signifying the importance of continued research [66,70]. Based on skinfold cut-points by Lohman [53], highSES boys and girls were more often classified as overweight or at risk for overweight than the low SES group. This may reflect differences in growth patterns, maturation timing, or structured lifestyle routines associated with socioeconomic context. It is advised to use a combination of body composition parameters, such as skinfold thickness, skeletal muscle, and WC, to determine BFP in children and adolescents [65,66].

In contrast with our study, Rönnecke et al. [66] found that girls had a slightly higher BFP than boys from the age of 6. In the current study, this is first observed at age 9, when girls from highSES groups have higher BFP (Figure 6a–d). For low SES participants, similar values are observed until age 9. Using Laursen et al.’s [71] cut-points and Lohman and Going’s [55] health-risk classifications (85th percentile = overweight; 95th percentile = obesity), children from high SES were more frequently classified in higher BFP percentiles (overweight) than those from low SES, particularly at age 9 (Table 1). This may reflect lifestyle-related factors, including greater access to energy-dense foods and increased sedentary behaviour. The study by Rönnecke et al. [66] primarily included a Caucasian sample from Germany, which may explain the slight differences observed in the ethnically diverse cohort in the current study. However, these metrics primarily serve as reference tools rather than validated predictors of cardiometabolic disease [66]. Our study yielded results similar to those of McCarty et al. [65] and Zborilová et al. [72], who reported on European children from the UK and the Czech Republic, and concurred that typically developing boys at the 50th percentile reach peak BFP values around the age of 11. However, this was observed only in the high SES group, as the low SES group reached peak values before age 10. During puberty, these changes in body composition are expected and serve as markers of maturation [73]. Additionally, being overweight is directly associated with earlier maturation [26].

#### 4.1.5. Skeletal Muscle

At age 6, low SES boys and girls had skeletal muscle mass below the 10th percentile cut-points by McCarthy et al. [65], while high SES children were mostly at or above the 25th percentile (Table 1). By ages 9 and 12, most children fell within the 25th–91st and 75th–98th percentiles, respectively. As McCarthy et al. [65] provide no clinical cut-offs, these values are descriptive only. Skeletal muscle exhibited a three-way interaction, resulting in distinct growth curves by age, gender, and SES (*p* < 0.01). Increases in skeletal muscle agreed with other international studies (Figure 7a–d) [72]. However, distinct growth trajectories were observed among girls from high and low SES backgrounds in our study. Both groups showed a decline in skeletal muscle percentage from age 12 onward, resulting in comparable values (Table 3). Although SES groups of boys showed relatively similar growth trajectories (Figure 7c,d), those from low SES at the 50th percentile had lower skeletal muscle compared to the high SES group. Nonetheless, this disparity lessens as children age. The observed higher muscle mass in older children may reflect normal growth patterns, differences in physical activity or nutrition, or population-specific genetic factors rather than implying elevated health risk. The higher values observed in older South African children highlight the need for future investigation into population-specific reference standards rather than direct application of UK norms.

#### 4.1.6. Waist Circumference (WC)

WC has been proposed as a practical indicator of central adiposity in children and may provide additional information beyond BMI for health-related monitoring. However, it is primarily intended for screening rather than diagnostic health risk classification [74]. In this regard, WC is one of the AHIs that has received attention only in recent decades.

In paediatric populations, a WC at or above the 90th percentile for age and gender is often used to indicate elevated central adiposity and associated cardiometabolic risk [75]. WC, used to screen for central obesity, was compared with age- and sex-specific NHANES III percentiles [76]. This study found that boys and girls from low and high SES groups were within the normal range across all ages (Table 1). Boys from low SES fell between the 10th and 25th percentiles, while girls from low SES fell between the 25th and 50th percentiles (Table 1). Boys and girls from high SES fell between the 50th–75th percentiles.

WC showed a three-way interaction among age, gender, and SES (*p* < 0.05), as well as two two-way interactions with SES (age, *p* < 0.01; gender, *p* < 0.05). Consistent with our findings, Rönnecke et al. [66] reported that 3- to 16-year-old German boys have higher WC values than girls, particularly after age 13. However, this was only the case for the high SES groups from the current study (Figure 8a–d). Frediksen et al. [74] and Mushtaq et al. [77] report that girls aged 6–12 in Norway and Pakistan typically have WC values similar to those of boys, even though they may have higher BFP values. Mushtaq et al. [77] reported findings similar to ours, concluding that WC is higher among individuals of high SES. This could be attributed to Pakistan’s socioeconomic structure being similar to SA’s. Although international age and gender cut-points for determining central obesity in children aged 6–18 are available, it is recommended that countries develop their own cut-points [75].

Limited data are available on AHI cut points across SES groups, making comparisons among them difficult. Although the current study found various noteworthy gender and SES differences in these AHI, results can only be compared globally as a combined group using universal cut-points. Although several studies from similar landscapes concurred with our findings on SES differences, these results remain inconclusive, as more in-depth research is needed to confirm these trends across the entire group. As highlighted in previous studies, deviations from age- and sex-specific percentiles should therefore be interpreted as markers of altered body composition or developmental trajectories rather than direct evidence of cardiometabolic or developmental risk [65,66,72,73,74,77,78].

### 4.2. Comparing National Growth References to Universal Cut-Points

In this context, the suitability of international growth references was evaluated based on the consistency of percentile distributions, alignment of *z*-scores, and the extent to which observed deviations reflected expected population variability rather than indicating clinically meaningful differences. Systematic deviations from previous research indicate that differences of approximately 0.5 SD and above the WHO standards may signal that international references do not fully capture local growth patterns, supporting the need for age- and population-specific growth curves [79,80].

#### 4.2.1. World Health Organization’s (WHO) Standards as a Benchmark for the Total Study Population

Compared with WHO standards at the 50th percentile, the entire group had slightly higher values (50th–75th percentiles) than the WHO median references at most time points for all three AHI (height, body mass, and BMI) (Table 4). Initial research to detect secular changes also detected growth differences within populations, attributed to influences such as genetics, ethnicity, SES, and environmental conditions [25,31,78]. The Dutch and Asian nations are considered among the tallest and shortest populations in the world, which has led countries to develop population-specific growth references for their own populations [78,81]. In the case of the Netherlands, children consistently exceed the WHO height standards, while Chinese children are generally shorter and heavier than the WHO curves suggest [78,81,82]. These differences are often attributed to genetic and environmental factors, underscoring the need to use national growth charts for accurate health monitoring [23,78]. Ethnic differences must be considered when applying the WHO standards, as they may lead to an erroneous underestimation of short stature due to genetic variability [78].

Similarly, Abdulrazzaq et al. [21] also reported that Emirati children were significantly shorter than the WHO standards. A wealthy country with access to quality food and healthcare sources, such as the United Arab Emirates (UAE), would be expected to exhibit similar statures to other developed countries like America [20]. Even children from high SES in Hong Kong were below the WHO standards compared to European children, who were significantly taller compared to global standards [18,20]. However, genetic variability between populations accounts for 24% of the genetic variance in height. Greater variability in stature is found among ethnic groups from the sub-Saharan African continent compared to those in Europe [81]. Historically, this variance has been underestimated [20].

Several countries compared their national findings with international growth standards to determine whether a true population-specific representation was reflected, given the diversity in genetics and SES within countries [21,22,25,32]. A Turkish study reported that children from the age of 10 are significantly taller compared to the WHO references [23]. Our study reported similar results, confirming taller stature in the total group from age 6 onward, with girls and boys being 1–2 cm and 2–3 cm taller, respectively, at each time point. National reference data include apparently healthy participants from diverse socioeconomic backgrounds and environmental conditions, indicating both potential growth impairment and optimal growth trajectories [24,82]. This provides a true representation of *“how the specific population is growing”* compared to WHO standards, which reflect *“how the population should grow”* [83]. Therefore, it is proposed that WHO standards be used to compare and benchmark national population growth with optimal growth for intervention purposes [1,23,82]. Chinese children were not included in the reference sample used to develop the WHO standards. The sample was not excluded for potential genetic variation in stature, but rather because China declined the invitation to participate [83]. Hence, the shorter average stature of Chinese children did not influence the WHO growth standards, as they were not included in the reference population. Therefore, the WHO charts remain a suitable and unbiased reference for comparing the growth of South African children when the sample represents typically developing children growing under conditions that approximate optimal health and environmental circumstances, as they have not been influenced by specific population-level genetic characteristics, such as those in China. The WHO charts offer a neutral comparison benchmark; however, this does not negate the importance of developing locally relevant growth data. Given that the total group consistently fell between the 50th and 75th percentiles for height at most time points, a slightly elevated BMI was expected. However, these values remain within the normal reference range and therefore reflect population-level variation rather than clinical growth abnormality at the individual level. This raises an important question: Are our children truly overweight, or could their higher BMI reflect increased height, potentially leading to misclassification of their weight status?

#### 4.2.2. Impact of Socioeconomic Status (SES) and Gender in Relation to World Health Organization (WHO) References

Additionally, our study found gender and SES disparities, confirming that girls from lower SES groups are shorter than those from higher SES groups (Table 1). However, findings suggest that boys from high SES appear taller than the WHO standards at age 6, but the gap gradually lessens as they grow, aligning more closely with the WHO growth curves over time. Compared to WHO standards, surprisingly, children from low SES are mostly aligned or slightly below these percentiles (25th–50th percentile). Thus, individuals from high SES exceed the WHO percentiles, particularly at the 97th percentile (Table 1 and Table 4). In particular, the high SES groups (boys and girls) exceed the WHO reference values, with higher BMI values at most time points. Boys and girls from low SES slightly exceed the WHO reference values for BMI at age 12, although at earlier ages they fall below the standard (between the 25th and 50th percentile). More longitudinal research during adolescence is needed to confirm these trends.

Optimal child growth can only be expected when generations have been living free from any environmental and dietary constraints [1,20]. From a South African perspective, households are experiencing a DBM where a high number of family members within one household (70.2%) are confronted with stunting in children and overweight/obesity in adults. However, the current study found that even those from low SES, who may be susceptible to poor health outcomes and food insecurity, can achieve growth standards (25th–50th percentiles) comparable to WHO standards, which are based solely on a selected sample not exposed to adverse environmental conditions. This can be attributed to the inclusion of various countries, which provide a representative sample that also faces significant SES disparities [14]. Among Brazilian children, both boys and girls from private schools (high SES) were significantly taller at most ages growing up compared to children from public schools (low SES) [21]. These findings from the Brazilian population provide a true representation of growth patterns in a developing country [32]. Similarly, the current study also reported higher body composition values for most AHI in children from high SES compared to low SES. Results from the Brazilian study confirmed that the weight and body mass of children representing a low SES area were not only lower than those of children from high SES areas but also lower compared to international standards [31]. However, in the current study, children from low SES were generally comparable to WHO references, particularly those at the 50th percentile. Silva et al. [32] confirm that growth references specific to low SES groups within a country are essential to monitoring growth in a diverse setting. The resulting effects, especially in stunted children, have detrimental long-term outcomes on labour productivity and ultimately, family income [11,84].

Comparisons with WHO 50th-percentile references highlight that children from high SES backgrounds consistently exceed the standards, often at or above the 75th percentile, corresponding roughly to deviations of +0.5–0.7 SD. In contrast, low SES groups tend to align with or fall slightly below the WHO medians, roughly −0.67 to 0 SD (25th–50th percentiles) across most age groups. These findings support the consideration of local, age-, gender-, and SES-specific reference curves for South African children, which would better reflect population variability and health risks. These findings suggest the need for further investigation due to differences exceeding the 0.5 SD threshold, particularly to elucidate the roles of genetic and environmental influences. It is also important to consider the context of the South African population, in which dietary patterns, such as a protein-rich diet, may contribute to these growth trends.

However, specific trends regarding gender, age, and SES emerged from the findings that warrant further in-depth, especially longitudinal, research on these sociodemographic factors, as well as on a much larger South African sample, since the study confirmed that children from higher SES exceed the WHO references at most time points. Although LMICs were included in the WHO references, it was still unexpected that children from low SES would be on par with, and in some cases surpass, the 50th percentile of the WHO standard. Although some groups appear to exceed WHO standards by more than 0.5 SD in several body composition measures, these deviations likely reflect population-level variation rather than evidence of inappropriate growth or misclassification, as the study did not utilise raw WHO data or perform formal inferential testing and is based solely on descriptive comparisons. This raises further questions about ethnic variation among South African children compared with other populations. More research is needed to confirm this phenomenon.

### 4.3. Limitations and Recommendations

This study is not without limitations. Future research should include a population-representative sample and annual measurements beyond the primary school years to better understand growth trajectories. Assessments of secondary sex characteristics were not included in this study, which limits our understanding of the timing and tempo of puberty and the onset of growth spurts. Skinfold thickness could not be directly compared, as other studies used skinfold sites such as the triceps, biceps, and subscapular as stand-alone sites to develop percentiles. In contrast, our study used the sum of skinfolds. This study did not include the waist-to-hip ratio (WHR) as an AHI, which is recommended for future studies to compile a comprehensive growth profile. Additionally, the study could not determine statistical significance between the WHO and the current cohort, as the authors did not have access to the raw data from the WHO sample. SES influences as a causal driver in the absence of household-level data should also be interpreted with caution.

Nonetheless, the study’s strength is that it significantly contributed to the new literature by exploring growth trends across various anthropometric assessments, being the first of its kind in SA and, more broadly, globally, as no other studies have conducted SES comparisons in this regard. The study further compared trends in South African children with universal growth standards to clarify the need to establish national growth curves and to identify at-risk child populations. A key strength of this study is the use of LMS curve modelling, which allows the construction of smooth, age-specific growth percentiles even with relatively small subgroup sizes. Smoothing the centiles effectively increases the sample information by adding strength across ages, improving precision at both median and extreme percentiles. The anthropometric data were also normally distributed, which further supports the robustness of the percentile estimates derived using LMS modelling. While this study benefits from a longitudinal design and includes children from low, medium, and high socioeconomic backgrounds in the North-West Province, the findings are based on a single province and may not fully capture the national diversity of South African children in terms of ethnicity, urbanisation, nutrition, and other socioeconomic factors. Further exploration of these trends is recommended on a much larger, nationally representative sample. As a deviation of ±0.5 SD is the accepted threshold for developing national growth charts, the positioning of our children at approximately −0.67 to 0 SD (between the 25th–50th percentiles) and 0 to +0.67 SD (50th–75th percentiles) indicates that growth patterns exceed this benchmark. Applying current design principles to South Africa’s youth population (with approximately 5.1 million children aged 5–9 years and 5.4 million aged 10–14 years [85] and stratifying by age (6–13 years), sex (male and female), and SES (low vs. high) would require roughly 200–1500 children per age, sex and SES subgroup to achieve precise and reliable smoothed centile curves [85,86]. Most growth studies focus on infants and children younger than 5 years, underscoring the need for further investigation of older children, particularly during their school years. Comparisons with older studies should be made with caution, as many of these studies used weight-for-age to define underweight in children aged 5 years and older. The WHO AnthroPlus software (updated in 2014, Version 1.0.4.) provides BMI-for-age (thinness) as the standard indicator for children aged 10 years and older. As such, minor discrepancies may arise when comparing our results with those from studies that used the older weight-for-age approach. An additional strength of the study is the inclusion of a diverse SES sample, which provides a representative sample of South African schoolchildren and supports reasonable generalisability within the South African context. The current study, limited to the NWP, should therefore be regarded as a foundational or pilot investigation that informs the design and scope of a future national growth reference study.

## 5. Conclusions

This study provides the first longitudinal description of multiple anthropometric and body composition trajectories among South African primary school children across gender and SES. While WHO growth standards remain appropriate for general growth monitoring in children aged 6–12 years, they are limited in supporting the context-specific interpretation required for clinical screening and early risk identification in South African children. Reliance on BMI-for-age alone does not distinguish normal gender- and SES-specific growth patterns from emerging excess adiposity or cardiometabolic risk. The lack of national reference data for complementary indicators such as WC, skeletal muscle, skinfold thickness and BFP, therefore, limits decision-making regarding follow-up and intervention. Notably, WC emerges as the most prominent and practical indicator, given its strong correlation with central obesity. National age-, gender-, and SES-specific reference charts would complement WHO standards and improve locally relevant growth assessment.

## Figures and Tables

**Table 1:** Descriptive sociodemographic, body composition and Z-score indices over a 6-year follow-up (*N* = 349).

	Boys (*n* = 165)						Girls (*n* = 184)					
	Low SES (*n* = 83)			High SES (*n* = 82)			Low SES (*n* = 118)			High SES (*n* = 66)		
	M ± SD	Min	Max	M ± SD	Min	Max	M ± SD	Min	Max	M ± SD	Min	Max
**Biographical information**												
**Age (years)**												
6 years old	6.76 ± 0.48	6.00	7.80	6.90 ± 0.55	6.10	7.80	6.70 ± 0.44	6.00	7.80	6.88 ± 0.54	6.10	7.70
9 years old	9.84 ± 0.38	8.99	10.67	10.04 ± 0.35	9.32	10.68	9.77 ± 0.37	9.00	10.68	10.01 ± 0.36	9.24	10.64
12 years old	12.84 ± 0.39	11.89	13.68	13.05 ± 0.35	12.34	13.68	12.78 ± 0.36	12.03	13.68	13.02 ± 0.36	12.26	13.65
**Body composition**												
**Height (cm)**												
6 years old	116.99 ± 4.96	102.10	130.30	124.50 ± 5.89	112.20	141.70	117.06 ± 4.94	107.90	128.50	122.33 ± 6.01	109.70	134.70
9 years old	132.33 ± 4.99	117.00	147.10	139.77 ± 6.60	123.20	161.00	134.03 ± 6.00	121.40	151.00	138.91 ± 7.84	113.00	155.00
12 years old	147.78 ± 7.15	131.90	169.40	157.24 ± 8.34	139.40	178.80	151.98 ± 6.38	136.70	168.80	157.35 ± 7.37	143.60	185.90
**Body mass (kg)**												
6 years old	20.67 ± 2.52	16.40	30.50	26.76 ± 5.73	17.20	45.90	20.74 ± 3.06	15.08	29.70	24.18 ± 4.97	16.40	42.80
9 years old	28.58 ± 5.26	19.60	55.90	37.31 ± 9.86	22.60	65.60	30.13 ± 6.88	21.70	55.80	36.09 ± 8.78	22.20	64.70
12 years old	39.05 ± 9.31	23.10	84.80	52.35 ± 14.17	30.80	100.80	44.17 ± 10.05	28.80	78.50	52.19 ± 12.37	36.90	95.20
**BMI (kg/m^2^)**												
6 years old	15.18 ± 1.46	12.90	22.90	16.50 ± 2.70	12.90	26.80	15.10 ± 5.00	12.70	20.70	16.10 ± 2.50	12.90	24.00
9 years old	16.27 ± 2.37	12.90	25.83	19.00 ± 3.90	13.30	30.80	16.70 ± 3.00	13.10	27.40	18.50 ± 3.80	14.20	29.70
12 years old	17.81 ± 3.23	14.00	32.00	21.10 ± 4.70	14.90	36.50	19.00 ± 3.60	13.90	33.70	21.10 ± 4.40	16.10	34.50
**∑Skinfolds (mm)**												
6 years old	12.40 ± 3.40	8.30	27.00	16.32 ± 7.54	8.25	47.25	15.04 ± 4.69	8.25	37.50	18.95 ± 7.44	9.00	40.50
9 years old	13.30 ± 7.30	7.00	57.50	21.85 ± 10.64	8.50	52.75	17.96 ± 8.49	7.50	46.50	24.83 ± 10.66	12.25	53.75
12 years old	15.10 ± 11.40	7.50	78.30	24.66 ± 15.10	9.00	76.00	21.62 ± 9.91	9.00	59.50	28.42 ± 13.51	14.00	74.75
**BFP (%)**												
6 years old	14.60 ± 3.30	8.70	27.80	18.10 ± 6.03	10.44	38.60	14.36 ± 4.02	7.59	29.09	17.36 ± 5.53	8.42	31.81
9 years old	18.10 ± 6.70	5.00	38.40	22.97 ± 8.44	7.10	42.50	18.86 ± 8.16	5.40	43.60	23.80 ± 8.67	9.80	46.80
12 years old	17.30 ± 7.10	7.60	41.80	22.00 ± 8.86	5.20	44.60	23.34 ± 7.85	10.10	46.50	26.79 ± 8.33	11.40	47.60
**Skeletal muscle (%)**												
6 years old	22.83 ± 3.80	12.10	31.60	27.60 ± 3.90	17.00	36.40	25.90 ± 3.10	17.70	33.40	27.70 ± 2.70	20.80	32.80
9 years old	30.24 ± 3.03	13.70	36.10	32.00 ± 3.00	20.70	39.60	30.30 ± 2.60	20.50	43.80	30.80 ± 2.60	24.00	35.80
12 years old	35.90 ± 4.93	13.90	63.20	35.91 ± 3.40	26.30	43.60	32.70 ± 3.10	14.30	37.50	32.50 ± 3.00	25.30	38.10
**WC (cm)**												
6 years old	52.06 ± 3.30	45.10	62.00	57.42 ± 6.90	47.00	84.00	52.49 ± 3.50	45.00	63.80	55.76 ± 6.11	48.40	78.20
9 years old	57.40 ± 5.20	49.10	81.50	64.53 ± 8.85	50.80	94.20	57.23 ± 6.14	48.00	78.90	61.72 ± 7.88	51.40	88.90
12 years old	62.20 ± 7.60	51.20	93.80	70.66 ± 10.28	54.50	99.40	63.61 ± 7.23	51.30	89.60	67.60 ± 9.82	54.80	103.10
**Z-score indices**												
**HAZ**												
6 years old	−0.69 ± 0.84	−3.21	1.53	0.52 ± 1.12	−2.28	4.81	−0.42 ± 0.85	−2.65	1.95	0.32 ± 1.11	−2.03	2.70
9 years old	−0.73 ± 0.79	−2.90	1.39	0.29 ± 1.03	−1.63	3.96	−0.50 ± 0.90	−2.60	1.66	0.04 ± 1.19	−3.89	2.70
12 years old	−0.97 ± 1.00	−3.61	1.73	0.12 ± 1.14	−2.53	2.43	−0.47 ± 0.92	−2.78	1.87	0.15 ± 1.08	−1.86	4.29
**WAZ ^**												
6 years old	−0.67 ± 0.87	−2.60	1.93	0.64 ± 1.40	−2.28	54.81	−0.42 ± 0.91	−2.71	2.02	0.35 ± 1.12	−2.17	3.59
9 years old	−0.36 ± 0.97	−3.17	2.96	0.50 ± 1.13	−1.63	3.96	−0.32 ± 1.00	−2.23	2.81	0.23 ± 0.94	−2.18	3.30
**BAZ**												
6 years old	−0.35 ± 0.86	−2.28	2.91	0.43 ± 1.49	−2.22	5.03	−0.28 ± 0.90	−1.97	2.49	0.17 ± 1.27	−1.82	3.17
9 years old	−0.29 ± 1.14	−2.76	2.94	0.84 ± 1.42	−2.37	4.24	−0.16 ± 1.14	−2.14	3.04	0.54 ± 1.24	−1.38	3.34
12 years old	−0.47 ± 1.36	−6.27	3.25	0.70 ± 1.44	−3.58	3.64	−0.11 ± 1.22	−2.61	3.03	0.50 ± 1.17	−1.97	3.12

BAZ = BMI-for-age; BFP = body fat percentage; BMI = Body Mass Index; cm = centimetre; HAZ = Height-for-age; kg = kilogram; M = Mean, Max = Maximum value; Min = Minimum value; SD = Standard deviation; mm = millimetre, SES = Socioeconomic status; ∑Skinfolds = sum of subscapular + triceps skinfolds; WC = waist circumference; WAZ = weight-for-age z-score; ^ = z-score omitted at age 12 due to biological changes in children older than 10 years old.

**Table 2:** Significant interaction effects of age, gender, and SES on AHI (*N* = 349).

Variables	Three-Way Effects		Two-Way Effects					
	Age and Gender & SES		Age and Gender		Age and SES		Gender and SES	
	F(df)	*p*	F(df)	*p*	F(df)	*p*	F(df)	*p*
Height	2(690) = 2.52	<0.081	2(690) = 26.58	<0.010 *	2(690) = 4.64	<0.010 *	1(345) = 5.241	0.023 *
Body mass	2(690) = 1.70	0.183	2(1035) = 0.48	0.953	2(1035) = 0.04	<0.965	2(1035) = 0.00	0.945
BMI	2(1035) = 0.01	0.993	2(690) = 5.96	<0.010 *	2(690) = 20.96	<0.010 *	1(345) = 1.65	0.200
∑Skinfolds	2(1035) = 0.01	0.993	2(1035) = 0.78	0.459	2(1035) = 0.71	0.931	2(1035) = 0.01	0.940
BFP	2(1035) = 0.41	0.667	2(1035) = 0.21	0.814	2(1035) = 0.92	0.398	2(1035) = 0.26	0.635
Skeletal muscle	2(663) = 5.30	0.010 *	2(663) = 69.39	<0.010 *	2(663) = 32.96	<0.010 *	2(343) = 6.53	0.011 *
WC	2(690) = 3.08	0.047 *	2(690) = 0.081	0.445	2(690) = 7.63	<0.010 *	1(345) = 4.278	0.039 *

BFP = Body fat percentage; BMI = Body Mass Index; SES = Socioeconomic status; ∑Skinfolds = sum of subscapular + triceps skinfolds; WC = Waist circumference; df = degrees of freedom; * *p* < 0.05 = Significant.

**Table 3:** Percentile cut-point for AHI at the 3rd, 50th, and 97th percentiles for gender and SES over 3 time point measurements.

AHI	Girls						Boys					
	Low SES			High SES			Low SES			High SES		
Age (Years)	3rd	50th	97th	3rd	50th	97th	3rd	50th	97th	3rd	50th	97th
**Height (cm)**												
6 years old	108.47	115.75	124.35	113.62	120.50	132.89	109.64	115.10	122.10	114.95	123.70	133.46
9 years old	124.08	132.80	143.44	124.88	135.95	152.03	124.63	130.85	138.77	129.66	139.80	148.10
12 years old	140.91	151.10	163.75	147.97	156.80	169.68	135.68	147.50	159.44	145.25	154.70	170.50
**Body mass (kg)**												
6 years old	16.36	19.65	26.44	18.81	21.30	35.41	16.75	19.80	25.70	18.37	24.70	33.50
9 years old	22.20	27.60	45.07	25.76	31.65	50.59	21.53	27.30	38.66	25.45	35.80	52.80
12 years old	30.06	42.00	63.88	38.58	49.40	79.01	28.39	38.00	53.92	32.92	47.70	78.56
**BMI (kg/m^2^)**												
6 years old	12.91	14.80	19.25	13.00	14.90	21.69	12.91	14.80	19.25	13.00	14.90	21.69
9 years old	13.23	15.70	23.85	14.37	16.93	27.64	13.23	15.70	23.85	14.37	16.93	27.64
12 years old	14.46	18.10	25.10	16.69	19.80	32.15	14.45	18.10	25.10	16.69	19.80	30.47
**∑Skinfolds (mm)**												
6 years old	9.25	13.50	24.86	10.01	15.50	28.70	8.50	11.63	18.26	8.57	13.75	27.29
9 years old	9.79	15.50	39.93	13.24	21.00	47.06	7.50	11.50	26.89	11.00	17.00	41.95
12 years old	10.75	17.25	43.38	14.48	21.00	58.24	8.13	12.63	24.72	9.79	20.50	55.24
**BFP (%)**												
6 years old	8.69	13.45	22.53	9.51	14.99	24.96	10.71	13.86	20.40	10.78	16.13	28.06
9 years old	6.42	17.40	35.19	10.69	21.15	39.38	8.02	17.70	32.45	8.13	20.50	37.92
12 years old	11.52	22.20	38.85	12.74	22.60	40.78	9.70	17.70	32.67	10.83	21.20	39.59
**Skeletal muscle (%)**												
6 years old	19.91	25.45	30.74	22.72	27.75	32.24	15.26	22.65	28.85	19.87	27.35	34.60
9 years old	25.92	30.20	33.54	25.49	31.70	34.81	24.12	30.25	33.81	28.83	32.35	36.90
12 years old	26.71	33.10	36.47	25.49	33.50	37.33	29.73	35.75	41.30	30.27	36.10	40.84
**WC (cm)**												
6 years old	47.00	51.50	58.23	49.50	52.80	67.10	46.55	52.45	58.39	48.10	56.80	74.46
9 years old	50.10	55.20	72.26	53.48	58.55	77.65	50.73	56.50	67.39	54.09	62.45	83.37
12 years old	53.29	62.00	78.61	58.07	65.50	88.23	55.02	61.00	73.56	56.01	71.90	89.12

BFP = Body fat percentage; BMI = Body Mass Index; cm = centimetre; kg = kilogram; ∑Skinfolds = sum of subscapular + triceps skinfolds; SES = Socioeconomic status; WC = Waist circumference.

**Table 4:** Comparison of the 3rd, 50th, and 97th percentiles of girls and boys with WHO AHI standards by timeline.

Age (Years)	3rd				50th				97th			
	WHO	NW-T	NW-L	NW-H	WHO	NW-T	NW-L	NW-H	WHO	NW-T	NW-L	NW-H
**GIRLS**												
Height (cm)												
**6**	105.50	109.68	108.47	113.62	115.13	117.50	115.75	120.50	124.75	130.76	124.35	132.89
**9**	121.00	124.10	124.08	124.88	132.49	133.60	132.80	135.95	143.99	146.40	143.44	152.03
**12**	138.37	141.49	140.91	147.97	151.23	152.95	151.10	156.80	164.10	167.57	163.75	169.68
Body mass ^ (kg)												
**6**	15.51	16.58	16.36	18.81	20.16	20.40	19.65	21.30	27.27	30.06	26.44	35.41
**9**	21.12	22.20	22.20	25.76	28.20	29.00	27.60	31.65	39.96	48.02	45.07	50.59
**12**	-	30.21	30.06	38.58	-	43.45	42.00	49.40	-	69.71	63.88	79.01
BMI (kg/m^2^)												
**6**	12.83	12.96	12.91	13.00	15.27	14.80	14.80	14.90	18.93	20.34	19.25	21.69
**9**	13.28	13.28	13.23	14.37	16.10	16.00	15.70	16.93	21.06	25.58	23.85	27.64
**12**	14.56	14.50	14.46	16.69	18.00	18.60	18.10	19.80	24.37	29.16	25.10	32.15
**BOYS**												
Height (cm)												
**6**	106.69	110.48	109.64	114.95	115.95	118.30	115.10	123.70	125.22	131.86	132.89	133.46
**9**	121.26	125.94	124.63	129.66	132.57	133.55	130.85	139.80	143.87	147.21	152.03	148.10
**12**	135.75	137.73	135.68	145.25	149.08	151.00	147.50	154.70	162.41	168.79	169.68	170.50
Body mass ^ (kg)												
**6**	16.11	16.87	16.75	18.37	20.51	21.30	19.80	24.70	26.66	31.74	25.70	33.50
**9**	21.60	22.50	21.53	25.45	28.11	29.35	27.30	35.80	38.56	51.48	38.66	52.80
**12**	-	29.07	28.39	32.92	-	41.10	38.00	47.70	-	73.27	53.92	78.56
BMI (kg/m^2^)												
**6**	13.16	13.27	12.91	13.33	15.31	15.30	14.80	16.00	18.29	20.32	19.25	20.19
**9**	13.61	13.58	13.23	13.77	16.05	16.21	15.70	18.15	20.11	24.27	23.85	26.69
**12**	14.60	14.90	14.45	15.16	17.53	17.90	18.10	20.80	23.05	30.21	25.10	29.90

^ No WHO reference data is available for weight beyond the age of 10 since height and body weight can be mistaken for growth increases during puberty. The WHO instead recommends using BMI. WHO = World Health Organization; NW-T = North-West Total group; NW-L = North-West low SES group; NW-H = North-West high SES group.

## Data Availability

If necessary, AP, the principal investigator of the research project, may be contacted to obtain the dataset.
